# Gravitational perturbations correlated with the asteroid kinetic impact deflection technique

**DOI:** 10.1038/s41598-022-15588-7

**Published:** 2022-07-09

**Authors:** B. S. Chagas, A. F. B. A. Prado, O. C. Winter

**Affiliations:** 1 Grupo de Dinâmica Orbital e Planetologia, São Paulo State University (UNESP), Guaratinguetâ, SP Brazil; 2 Academy Engineering, RUDN University Miklukho-Maklaya, Street 6, 117198 Moscow, Russia; 3 INPE: National Institute for Space Research, São Josẽ dos Campos, SP Brazil

**Keywords:** Astronomy and planetary science, Engineering

## Abstract

This paper presents the use of the kinetic impact technique to deflect asteroids that may present some risk of collision with Earth. Within the work to be developed here, we intend to evaluate in more detail the possibility to deflect the orbit of the asteroid 101955 Bennu by applying variations in its velocity ($\Delta$v) at different positions along its orbital period and measuring effects of close encounters with planet Earth. We will see that, in a relatively long period of time, the asteroid has several close encounters with the planet, thus suffering a natural gravitational perturbation. With the application of the impulses, the relative distances change, causing variations in the energy of the asteroid and a large variation in the relative distance between the asteroid and Earth over a long period after the impulse. We present results related to the magnitude of the impulse applied, which is important because its defines the mass and velocity of the impactor to be considered. Then, we mapped the positions of the impulses along a period of the orbit of the asteroid. We finish by explaining what happens to the orbit of the asteroid during the periods of gravitational perturbation, since the close encounters amount to “Swing Bys” that intensify the variations of the relative distances between the bodies after the impulse is applied.

## Introduction

Asteroids are small rocky or iron bodies that are remnants of the formation of the solar system^1^. These bodies can present great danger to life due to possible impacts with our planet. Studies show that, for land and ocean impact scenarios, their results can be catastrophic^2^. In addition, they can be sources of commercial and space exploration and also of scientific research. This problem has already been discussed in a range of publications^3^. We also run into the condition that these objects are generally very small compared to planets and moons and, due to this factor, they have a weak gravitational attraction, which allows irregular shapes for the bodies^4^.

Over the years, several proposals have come up with different techniques to make perturbations in the orbit of an asteroid and thus deflect it. Methods such as changing its velocity by the application of an impulse were proposed, as well as the use of nuclear resources, solar sails, solar radiation collectors and laser systems^5^. Carusi et al.^6^ also made studies related to the ‘distributed deflection’ technique, that can be used when it is known that an asteroid will collide with the Earth in a certain relatively long time (50 years was used) and that it passes through its perihelion many times before the impact. In this situation, successive $\Delta$v can be applied in its orbit, thus allowing the use of lower energy methods, such as the kinetic impact technique and also solving the problem of the mass of the possible impactor, where we can apply moderate impulses successive times, which can deflect even larger objects, as an example, we will be able to deflect objects 4 or even 5 times larger with the same energy used to deflect smaller objects^7^.

It is worth mentioning the case of objects that have a close encounter with Earth before impact. For these cases of resonant returns there is a lot of difference if the maneuver is performed before the last close approach, allowing a much larger object (1 km) to be deflected with the same energy used to deflect a smaller object (100 m)^8,9^. However, Carusi et al.^5^ showed that the occurrence of a resonant return will not always have the same effect from one case to another.

Ledkov et al.^10^ showed that the use of gravitational maneuvers of a small asteroid with the Earth can be used in the planetary defense scenario. In this work they propose that, through the application of small impulses in the orbit of a small asteroid along the approaches to Earth, a larger asteroid can be redirected to impact with a potentially dangerous asteroid. Boley and Byers^11^ warn about the planning of asteroid deflection missions in order to use prevention mechanisms to place an asteroid in a safe place, but also showing that it can happen that, due to the action of man, the asteroid can enter a keyhole, creating both beneficial results and new risks and that the precautionary mechanism should be well evaluated.

Ahrens and Harris^12^ developed a work relating the forms of deflection of the asteroid with the fragmentation generated by them. They showed that, for perturbations that could take decades, velocity variations of about 1 m/s would be enough to generate significant changes in the orbit of the asteroid. In this work they showed that asteroids close to Earth can be deflected by varying their velocity, both increasing or decreasing, in relation to the Sun. Some studies also discuss the fragmentation which occurs upon a too large impulse being imparted, or by natural impacts.^13–15^. They show that too large impulses should be avoided for a kinetic impact.

The kinetic impact deflection technique is gaining more and more priority in the scientific community, as it proves to be the most practical technique. The first real asteroid deflect mission is the DART mission, whose main objective is to quantify the deflection capacity of an asteroid using this technique^16^. The satellite called Dimorphos will be impacted by a spacecraft, causing its orbit to be disturbed and, consequently, the orbit of the main body Didymos. Perhaps the heliocentric orbit change can be resolved by orbital tracking of the Hera spacecraft, a mission in partnership with DART, and ‘coupling’ that to the Didymos system by camera and startracker.^17–19^.

When two or more bodies collide, there is a large set of possible outcomes, ranging from readjusting shape, size, outer surface, and rotational states. This analysis provides a detailed view of a number of mechanical processes that still need to be quantified, such as the formation of craters due to impact and the final velocity after the impulse is applied by the kinetic impact technique^20^.

Park and Ross^21^ showed that the change generated by an impulse is strongly dependent on the location where the impulse is applied in the orbit, as well as the direction of the impulse in relation to the orbital velocity. In this work, they also argued that the impulse applied at the perihelion is the best for alert times longer than an orbital period.

Conway^22^ showed that, if an impactor is launched less than a year in advance of a possible collision of an asteroid with the Earth, the deflection obtained will be on the order of the diameter of the Earth for each 1 m/s velocity change applied to the asteroid. It also points out that, considering the mass of larger asteroids, it would be very difficult to change its velocity by 1 m/s, thus warning that to be able to effectively apply the kinetic impact on moderate sized asteroids it is necessary to apply the impulse several years before a possible collision. If we have less than a year, it becomes more difficult to carry out the mission, as it would require a very large speed variation, which could fragment the asteroid.

Another study on disturbing an orbit of the asteroid using propulsion mechanisms coupled to the surface of the asteroid, paying attention to the disturbance of the rotation of the body, can be found in Fargion^23^. This work also mentions the use of gravitational perturbation variation to deflect asteroids, citing the asteroid Apophis as a good opportunity for this study.

An interesting proposal suggests using the electrostatic principle to generate the force needed to deflect asteroids. The proposal depends heavily on the generation of force provided by the interaction of a charged body with the electrostatic field, where an asteroid is considered a charged body that will be reacting with the electrostatic field generated by a dipole. The technique is presented by Gonzaga^24^, where he also presents the challenges for the use of this proposal^24^.

The proposal of Maccone, generates an interesting discussion about building two space bases at points L1 and L3 closest to the Earth-Moon system, since these two points do not change their distance from Earth. Missiles are able to reach orthogonality the impact trajectory of the asteroid, causing it to be deflected by 10000 km, a distance considered safe by the author. However, it is observed by the same author that this distance can be changed by more refined analyses. Maccone suggests using confocal conic trajectories for missiles to intercept an approaching asteroid at 90 deg angle. It is shown that, if the asteroid is not deflected at the first moment, we could fire more than one missile in succession until it manages to deflect its orbit, since the trajectories of the subsequent missiles are orthogonal to any hyperbolic trajectory that the asteroid may have^25–27^.

Izzo^28,29^ derived an analytic expression relating asteroid deflection, primarily in orbit phase or encounter time, to velocity change, either impulsive or long duration, and applied the expression to compare kinetic impactor and long duration thrusting approaches.

The objective of this work is to evaluate the kinetic impact technique to deflect the orbit of potentially dangerous asteroids in relation to Earth considering the perturbations of the planets in the solar system and also how this relationship is made over a period of approximately 100 years after the impulse is applied to the asteroid. So, we will vary the velocity of the object along its orbital period, as done in Negri et al.^30^. The asteroid chosen is the asteroid 101955 Bennu, which is part of the group of asteroids that have a near-Earth orbit (NEAs). This asteroid was a recent target of the Osiris Rex mission of NASA, gaining even more visibility within the scientific community. But the technique used here can be used for other targets such as the asteroids 99942 Apophis^31–34^, 65803 Didymos^19,35–37^, 1950 DA^38–40^, among other PHAs. We will map positions along the orbital period of a dangerous asteroid to find the best deflection scenarios of an asteroid considering a longer time span of 100 years (Figs. 2, 3 and 4), where, we will show that the various approaches of the asteroid over time with the Earth will act as swing bys, making the asteroid to move forward or delay its Earth encounters, and making it approach closer or farther from the Earth in future encounters.. This effect will be best shown in Fig. 11.

## Methods

In this work, we will analyze the gravitational effects involved in the kinetic impact asteroid deflection technique over a relatively long period. In this process we will solve the N-body problem using the Mercury integrator package^41^, considering the Bulirsch-Stoer integrator. We are considering all the planets in the solar system, the Moon and the target asteroid for our study, with the Sun as the central body.

The equation of motion for the N-body system with a body of mass much larger than the others can be written as follows^42^

1 $$\frac{{d^{2} \left( {\vec{r}_{i} } \right)}}{{dt^{2} }} + G\left( {m_{n} + m_{i} } \right)\frac{{\vec{r}_{i} }}{{r_{{in}}^{3} }} = G\sum\limits_{{j = 1,j \ne i}}^{{N - 1}} {m_{j} } \left( {\frac{{\vec{r}_{j} - \vec{r}_{i} }}{{r_{{ij}}^{3} }} - \frac{{\vec{r}_{j} }}{{r_{{jn}}^{3} }}} \right){\text{ }}$$

where $\mathbf {r_i}$ and $\mathbf {r_j}$ are the position vectors of particles i and j in relation to the central body, G is the gravitational constant, $m_n$ is the mass of the central body, $m_i$ and $m_j$ are the masses of particles, $r_ {in}$ and $r_ { jn}$ are the distances between particles i and j in relation to the central body and $r_ {ij}$ is the relative distance between particles. The bodies used in the simulation will be presented later.

With the evolution of the research, we realized that our results had a larger contribution of maneuvers assisted by gravity, ‘swing by’, which proved to be the main cause of changes in the orbit of the asteroid. ‘Swing by’ maneuvers have been known for a long time in the astronomical scene and therefore are not new. A description of this maneuver can be found in Prado^43^ and Ferreira et al.^44^.

### Development

For the input data for integration, the position and velocity components of the bodies, taken from the JPL Horizons website, were used. The mass of $(7,329 \pm 0,009)10^{10}$ kg and density of $(1190\pm 13)$ kg $m^{-3}$ were considered for the asteroid Bennu^45–47^. Cartesian coordinate values can be found in Tables 1 and 2. We are not considering the composition and dimensions of the asteroid, as well as its rotation properties.

First, we found the closest approach between the asteroid and Earth in a 100-year period from August 5, 2021, the date we began the study. All planets in the solar system, the Moon and the asteroid Bennu were included in our simulations.

This closest approach occurred on September 22, 2080, at a distance of approximately 0.005 au. From this date onwards we collected the positions and velocities of all bodies and performed a simulation going 100 years back in time, reaching the new starting point. Then, we entered the values of the new starting point and proceeded to perform simulations over time applying velocity variations to the initial data of the asteroid Bennu. It is important to mention that, when performing the process of using the initial data of the bodies extracted from the simulations in Mercury, going back in time and returning the simulations forward again from the new data obtained in the simulation, the bodies return in 2080 with the same orbital characteristics, with the same coordinates, velocities and relative distances. The variations of velocity are in the range -50 mm/s to 50 mm/s, with an increment of 10 mm/s. This methodology has already been used in many works found in the literature, such as Ahrens and Harris^12^, Carusi^5^, among others.

**Figure 1:**
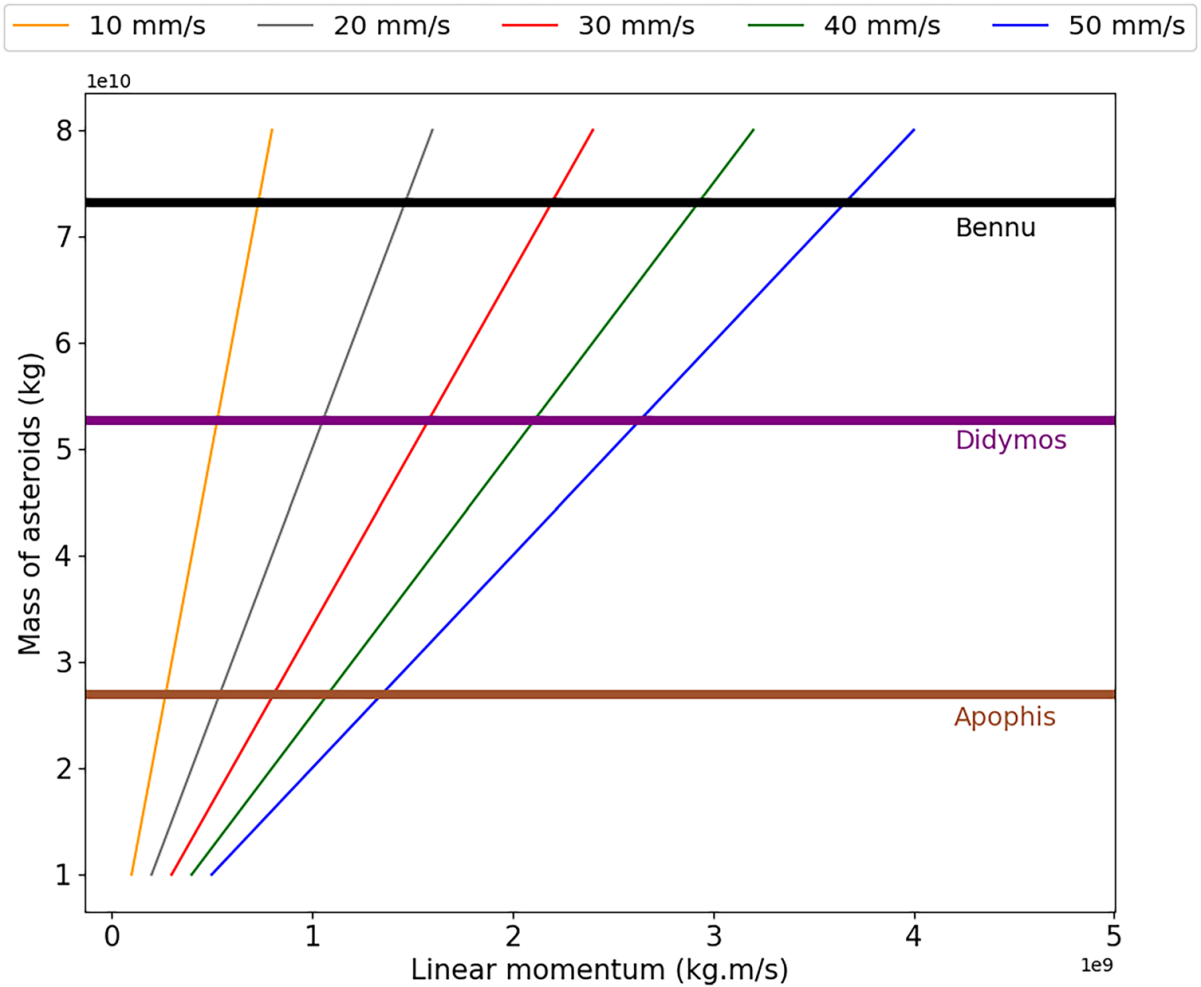
Relationship between the linear momentum required to make positive velocity variations in the asteroids as a function of the mass of the asteroids in a scale of $10^{10}$. The results for negative changes in velocity are symmetrically equal, having a negative momentum value. We are using the asteroids 99942 Apophis, 65803 Didymos and 101955 Bennu, the latter being the target of this work.

To exemplify the relationship of linear momentum required for a spacecraft to make each change in velocity, we are showing, in Fig. 1, the relationship of linear momentum to make the change in velocity of the asteroid as a function of the mass of the asteroid. We can see that the momentum increases linearly with the mass of the asteroid for each change in velocity, and that, for each asteroid, we must associate the magnitude of the impulse according to the mass of the impactor and the change in velocity that we will want to apply to the orbit of the asteroid. It is evident that, for larger impulses, we will need a larger impactor and this becomes a motivation to find better diversion conditions in order to enable the use of this technique.

To better understand the phenomenon, we chose to divide the orbit of the asteroid into 36 parts, to collect more information about the application of impulses on the asteroid in different positions of its orbit and thus be able to understand what is required for a possible future mission. To carry out this process, the simulations went back in time so that the impulses were applied at each position of the asteroid, with the difference that the positions are in intervals of approximately 10 in the mean anomaly of the orbit of the asteroid. Thus, the integration period increases a little more than the initial period, which was 100 years, and the orbital period of the asteroid in question is approximately 1.2 years.

The impulses were applied at the center of mass of the asteroid, and in the direction of its orbital velocity vector. Variations in this impact angle will be developed in future.

**Table 1:** Input values for the coordinates of each body as of August 5, 2021. Data taken from JPL NASA.

Bodies	x(au)	y(au)	z(au)
Mercury	$-2.896323130935082E-01$	$1.850577763255397E-01$	$4.169019903669655E-02$
Venus	$-5.692473108639921E-01$	$-4.448853077740749E-01$	$2.674283485411453E-02$
Earth	$6.855761418396654E-01$	$-7.477764241622177E-01$	$3.211115876042908E-05$
Moon	$6.855266149744332E-01$	$-7.451016724453864E-01$	$1.230222158011698E-04$
Mars	$-1.614905800650153E+00$	$3.955532232885259E-01$	$4.790302296698644E-02$
Jupiter	$4.148571639394127E+00$	$-2.841339576414095E+00$	$-8.101578326784213E-02$
Saturn	$6.390745065584204E+00$	$-7.624741172250260E+00$	$-1.217720468705376E-01$
Uranus	$1.479576471988037E+01$	$1.307144787474826E+01$	$-1.431624026194872E-01$
Neptune	$2.956816826343533E+01$	$-4.557431754532644E+00$	$-5.876364308100537E-01$
Bennu	$-8.178122041042245E-01$	$6.126618626692276E-01$	$6.774426832917149E-02$

**Table 2:** Input values for the velocity of each body as of August 5, 2021. Data taken from JPL NASA.

Bodies	vx(au/day)	vy(au/day)	vz(au/day)
Mercury	$-2.090746004400052E-02$	$-2.253446087272209E-02$	$7.638025951147821E-05$
Venus	$1.231235542811912E-02$	$-1.603266518952336E-02$	$-9.305216171721341E-04$
Earth	$1.240809069382479E-02$	$1.156282606438427E-02$	$-1.150024366950303E-06$
Moon	$1.184355116475734E-02$	$1.153246162528540E-02$	$4.458968850063871E-05$
Mars	$-2.807629223103034E-03$	$-1.239693158153517E-02$	$-1.909313502062999E-04$
Jupiter	$4.177326698222665E-03$	$6.587451407751826E-03$	$-1.208714010214003E-04$
Saturn	$3.969863463512147E-03$	$3.576887186643701E-03$	$-2.197897896266317E-04$
Uranus	$-2.628949090335184E-03$	$2.770739109958900E-03$	$4.429549807400130E-05$
Neptune	$4.618822543692893E-04$	$3.128329541906231E-03$	$-7.516594581186683E-05$
Bennu	$-1.309992480373342E-02$	$-1.191701206869430E-02$	$-1.210612917719958E-03$

### Ethical approval

The submitted work is original and not have been published elsewhere in any form or language. The work presents the results of a single study. The results are presented clearly, honestly, and without fabrication, falsification, or inappropriate data manipulation. No data, text, or theories by others are presented as if they were the author’s own.

## Results

**Figure 2:**
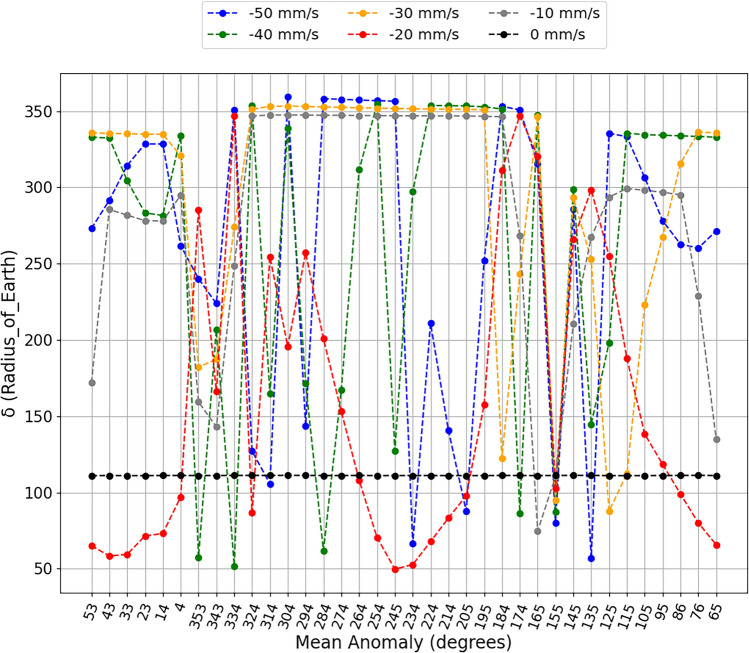
The closest approaches between the asteroid and the Earth along a period of about 100 years ($\delta$) (y axis), after negative velocity variations at different mean anomalies (x axis) to simulate different positions for the application of impulse. The black dots are the closest approach of the asteroid to the Earth without the application of impulse. The $\delta$ is shown in terms of Earth radii.

**Figure 3:**
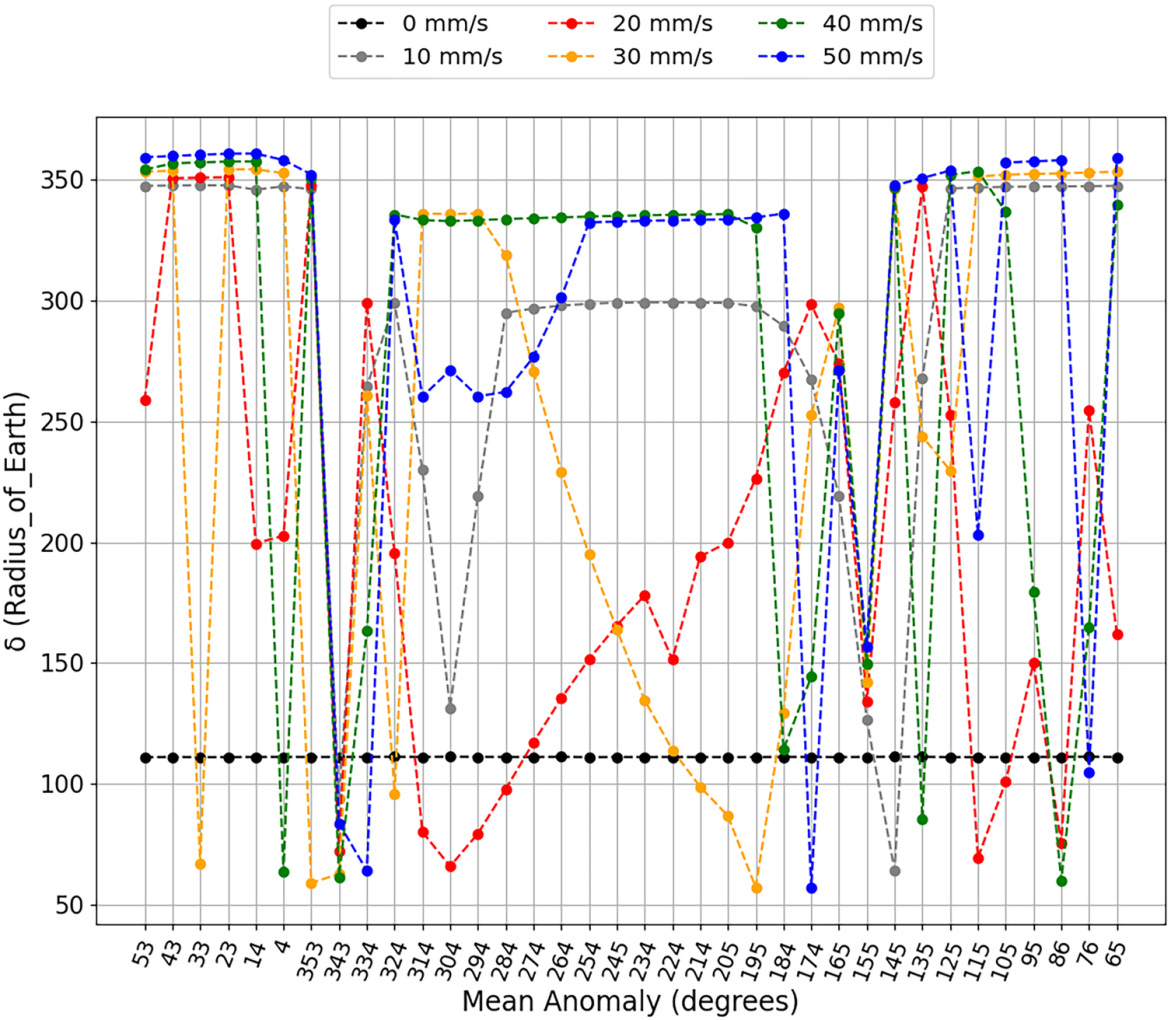
The closest approaches between the asteroid and the Earth along a period of about 100 years ($\delta$) (y axis), after positive velocity variations at different mean anomalies (x axis) to simulate different positions for the application of impulse. The black dots are the closest approach of the asteroid to the Earth without the application of impulse. $\delta$ is expressed in terms of Earth radii.

The simulations show us considerable variations in the closest approach between the asteroid and the Earth for each impulse applied at each different position. These results can be found in Figs. 2 and 3, where we separate the applications of negative variations in the velocity of the asteroid, when the impulses are applied opposite to the movement of the asteroid, as shown in Fig. 2, and for positive variations in the velocity of the asteroid, when the impulses were applied in the same direction of the movement of the asteroid, as shown in Fig. 3. These Figures show the closest approach (in units of radius of the Earth) between the asteroid and Earth found in the simulation period in the vertical axis and the applied impulse position (mean anomaly, in degrees) in the horizontal axis.

Looking at Fig. 2, it is interesting and encouraging to note that, for impulses applied at some positions of the asteroid, the smaller variations in velocity were almost equivalent to the larger applied impulses. This is encouraging as it shows that we will not necessarily need a large impactor to deflect the asteroid, but rather determine the best position for the intended objective for the asteroid to receive the impulse. For example, applying impulses at 25° from the aphelion of the orbit of the asteroid (mean anomaly 155°), all impulses against the motion of the asteroid bring the asteroid even closer to the Earth. The opposite effect occurs when the impulses are applied in the same direction of the movement of the asteroid, as we can see in Fig. 3, where all the impulses are applied in this same position to move the asteroid away from the Earth. However, for impulses applied close to aphelion (mean anomaly 184°), both scenarios moved the asteroid away from Earth, and this could be a safety region for the asteroid deflection scenario.

An interesting result shown in Fig. 2, to exemplify the idea of using smaller impulses to reduce the size of the impactor, which would facilitate the use of the kinetic impact deflection technique, would be for the impulse of −10 mm/s applied to the asteroid. We can see that, in just two situations this impulse brought the asteroid even closer to the Earth (mean anomalies 165° and 155°). If we observe the impulses applied between the anomalies of 324° and 184°, we see that it showed almost equivalent results to the largest impulses in the concept of moving the asteroid away and, in some positions, giving better results than larger impulses. Looking at Fig. 3, we found a similar scenario, changing only the positions where there is a closer approach between the bodies due to impulses of 10 mm/s. These impulses occur in anomalies of 343° and 145°, and the positions of greater distances between bodies occur between mean anomalies in the interval from 353° to 125°.

The same analysis can be performed by looking at the impulses applied near perihelion. Intending to follow the previous idea, by analyzing Fig. 2 we found that the impulse of −40 mm/s makes the asteroid to get closer to Earth when applied in the mean anomaly of 334°, 26° before perihelion, while when it is applied in the position closer to perihelion (mean anomaly of 4°), the impulse of −20 mm/s makes the asteroid to get closer to Earth. The same happens when the impulses are applied in the same direction of the motion of the asteroid. This can be observed in Fig. 3, where, for the same positions, the largest impulses are 40 mm/s and 50 mm/s, and they brought the asteroid even closer to Earth. It is clear that the magnitude of the impulse in this region is crucial to whether the asteroid will come closer at some point or if it will move away from the Earth. It shows that, for a scenario where we would be forced to apply the impulse in this region, the magnitude of the impulse must be considered independent of whether we are going to apply the impulse opposite to the movement of the asteroid or in the same direction of it. In a scenario of deflecting the asteroid away from the Earth, larger impulses are not always the best alternatives.

We believe that the results presented by the impulse of −20 mm/s, in Fig. 2, are also important, since this impulse brought the asteroid even closer to the Earth in different application positions. We also found some ‘jumps’ that occur when the impulse is applied at the average anomaly of 324° and 334°, where the asteroid is moving closer to Earth in the first case and farther from Earth in the second case. We will discuss a little more the results of −20 mm/s for these two positions to better explain the results presented here. Looking at Fig. 3, we see that, for the 20 mm/s impulse, situations are also presented in which the asteroid passed close to Earth when compared to other impulses applied. In these Figures, we notice that, for impulses of −20 mm/s and 20 mm/s, for each position, the results vary drastically.

In Fig. 2, there are some positions where all the impulses moved the asteroid away from the Earth, like for the mean anomalies of 95°, 105°, 145°, 184°, 195°, 274°, 294°, 304°, 343°. We also found positions in Fig. 3 where, in the mean anomaly positions of 14°, 23°, 43°, 53°, 65°, 95°, 125°, 155°, 165°, 184°, 224°, 234°, 245°, 254°, 264° and 274°, all impulses moved the asteroid away from the Earth. These are positions that would be good options to apply the impulse to move the asteroid Bennu away from the Earth. As we can see, large variations occur in the closest approaches between the asteroid and the Earth for impulses applied outside the perihelion and aphelion of the orbit of the asteroid around the Sun. Other cases can be discussed in future works.

Ferreira et al.^44^ showed that a satellite undergoing a propelled Swing By has the same characteristic of the results found here. Analyzing this work, we realize that we are working with the same idea, where we can employ the same explanation for the kinetic impact asteroid deflection scenario, where the impulses applied to the asteroid and the gravitational effect combine, so intensifying the variations in the orbit of the asteroid. We are just considering that this process occurs several times, generating an accumulation over time of the disturbances suffered by the asteroid and intensifying its energy variation.

Figures 2 and 3 give us interesting long-term ideas of the closest approaches between the bodies, and can serve as a guide to determine the best position to apply impulses in the orbit of a given asteroid. With that in mind, we can express them as Maps for Impulses in the Orbit of Asteroids (M.I.O.A.).

### Impulses in the perihelion and aphelion of the orbit of the asteroid

**Figure 4:**
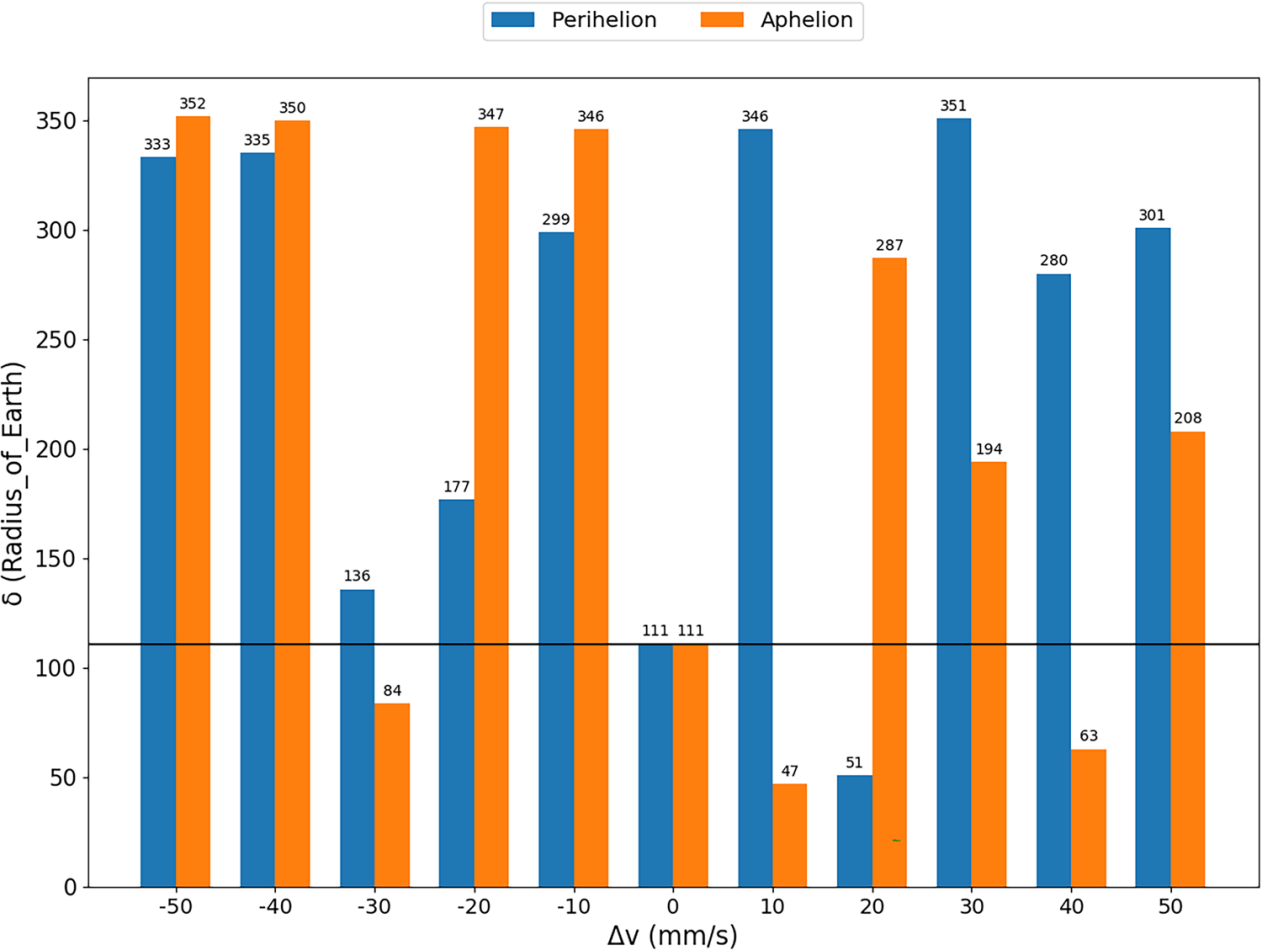
The closest approaches among the various approaches between the asteroid and the Earth ($\delta$) (y-axis), over the simulation period, after variations in velocity (x-axis) for the impulses applied at perihelion (blue) on July 19, 1980 and aphelion (orange) on December 11, 1979. The horizontal black line indicates the value of the closest approach of the asteroid to the Earth without the impulse. The exact value of the distance between the bodies is expressed above each bar.

Looking at the M.I.O.A., we notice that, by dividing the orbit of the asteroid into 36 parts, we did not completely reach the positions of perihelion and aphelion, but positions close to them, with anomalies of 4° and 184°, respectively. However, we find in the literature that the impulses applied in these positions must be considered^7,21^. Following this idea, we performed more simulations applying impulses in these two positions of the asteroid. The closest approaches between the asteroid and Earth for each impulse applied is shown in Fig. 4.

The black horizontal line represents the value of the closest approach of the asteroid to Earth without impulses being applied. We found that, for impulses applied at perihelion, only the impulse of 20 mm/s brought the asteroid even closer to the Earth, while for the impulses applied at the aphelion of the asteroid, magnitudes of −30 mm/s, 10 mm/s and 40 mm/s bring the asteroid closer to Earth, with the 10 mm/s impulse applied at aphelion and the 20 mm/s impulse at perihelion bringing the asteroid closer to the Earth within one mean Earth-Moon distance.

It is interesting to note that, for the smallest impulses, −10 mm/s and 10 mm/s, we achieved good results in terms of moving the asteroid away, as we have already seen in the previous subsection, with the exception already mentioned in the previous paragraph. The positive impulses (in the same direction of the motion of the asteroid), bring the asteroid even closer to Earth. We should also mention that the impulse of −20 mm/s, which for the mean anomaly of 4° moved the asteroid even closer to the Earth, when applied at perihelion is capable of moving it away. That is, a small difference in the point of application of the impulse is decisive to bring the asteroid closer or move it away from Earth.

Looking at Fig. 4, we also found that, for the impulses applied at the perihelion of the orbit of the asteroid, all negative impulses moved the asteroid away from Earth. Something to analyze would be the region already mentioned for impulses of −10 mm/s, which give the best results to move the asteroid away from Earth for this magnitude of impulse. We found that, by applying the −10 mm/s impulse in the aphelion of the orbit of the asteroid at approximately 36° before the perihelion, the asteroid will move farther away. This system has a complex dynamics, therefore it becomes difficult to make analytical predictions, which means that the only way to measure the effects is to perform numerical simulations, like the ones presented in this work, which justify the importance and necessity of the present work.

**Figure 5:**
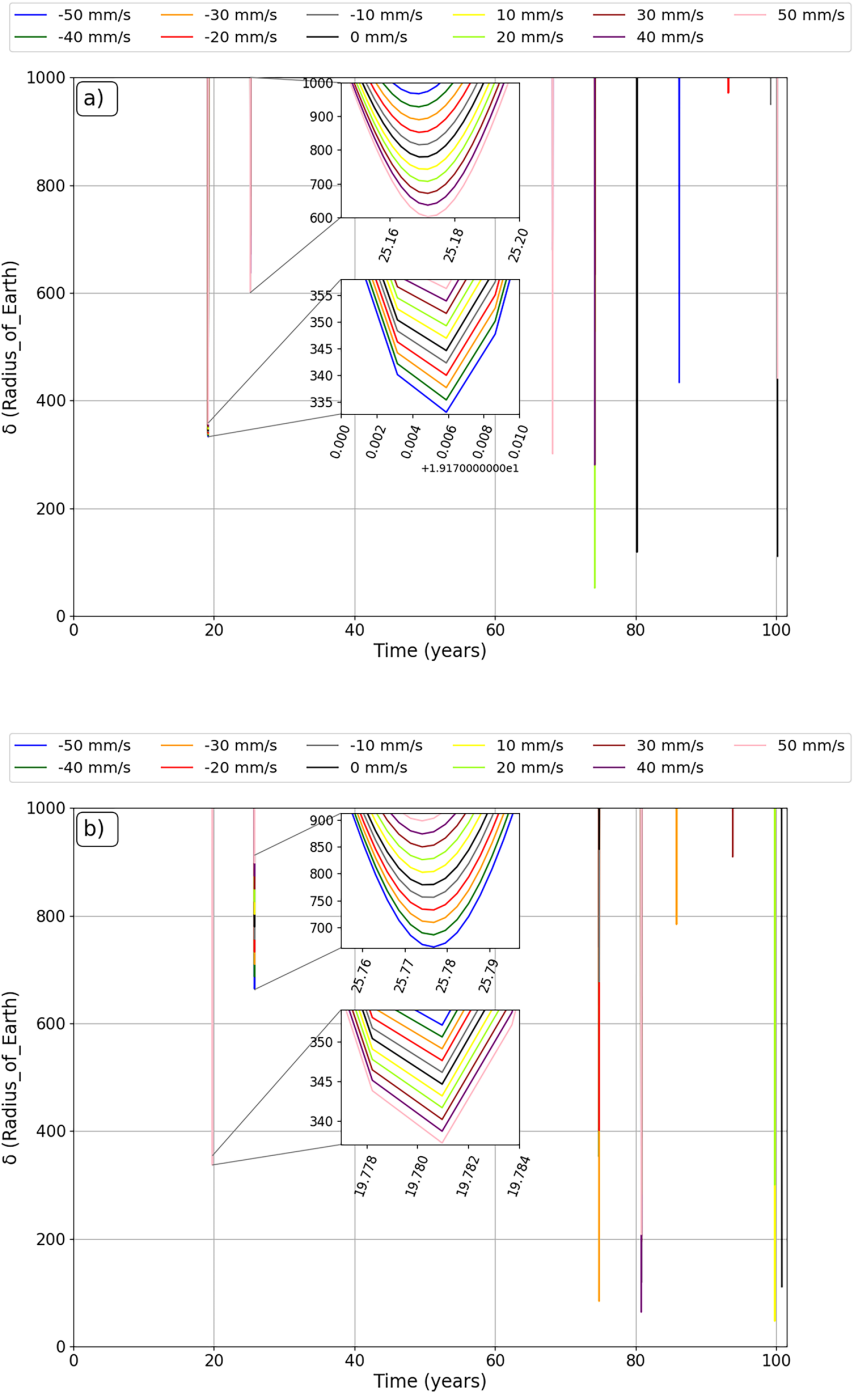
The relative distances between the asteroid and the Earth ($\delta$) for an impulse applied at the perihelion on July 19, 1980 (**a**) and aphelion on December 11, 1979 (**b**). We zoomed in on the first two close encounters to better visualize the variations in the approach between the asteroid and Earth.

Figure 5 presents the close approaches that occur between the asteroid and the Earth for the proposed simulation period, which is approximately 100 years. We limit the vertical axis, the approaches of the asteroid to the Earth ($\delta$) in units of radius of Earth, to 1000 Earth radii to better visualize the approaches that occur over the period. We will have the impulses applied at perihelion in Fig. 5a and aphelion in the Fig. 5b. These Figures will help us to visualize what we have explained about Fig. 4.

We zoomed the first two approaches within the mentioned limit and realized that, in the first approach between 19 years and 20 years after the impulses is applied, variations in the approach distance are already noticeable, where we see that the magnitude of the variations were proportional to the impulses applied. The second approach makes the variations even more clear, intensifying the variations after the first approach, which indicates that something else is intensifying these variations and not just the impulses applied in the orbit of the asteroid, since the variations are much larger than expected for a not so long time span. If we consider the next approaches we will see that the phenomenon is even more visible.

So, we realized that the various planetary encounters of the asteroid are interconnected with its natural trajectory and that each close approach amplifies the initial small impulse applied. However, the perturbations do not always amplify. If the first fly-by makes the next one more distant to the planet, the effect of the 2nd fly-by is reduced. The product of both could go down, or even the direction of changes can be reversed. In other words, what really generates larger effects is the gravitational disturbance over the years to which the asteroid will be exposed after the impulse. The impulse ends up being the trigger to change the behavior of the orbit of the asteroid, however, the factor that is really responsible for considerably altering the orbit of the asteroid are the variations that occur with each new close encounter between the asteroid and the Earth and we realize that each impulse applied has its own characteristic.

An equally important result is related to the date when the new closest approaches occur after the impulse. Looking at Fig. 5a, we found that the asteroid will approach Earth even closer on September 23, 2054, for the impulse of 20 mm/s. From Fig. 5, we have the new dates where the closest approaches occur for the impulses of −30 mm/s, 10 mm/s and 40 mm/s, are May 2, 2055, May 1, 2080 and May 1, 2061, respectively, confirming the results obtained in Fig. 4.

### Impulses applied in the mean anomaly of 324° and 334°

In the previous subsections, directly related to Fig. 2, we mentioned some interesting regions where, when applying certain values for the impulse, the asteroid can move away or closer in a range of approximately 10 from the position of its application. This result is interesting, because it proves our idea of the importance of perfectly knowing the position of the impulse. We have already explained our interest in the impulse of −20 mm/s, where the impulse showed the largest number of scenarios where the asteroid approached Earth even closer and also the existence of a ‘jump’ between the impulses applied at the mean anomalies of 324° and 334°. This difference found in the two positions is the target of this section, since we found intriguing the fact that in one position the asteroid goes closer to the Earth and, in the next position, there is a great distance of about 346 Earth radii of the asteroid. We will dedicate this section exclusively to the impulse of −20 mm/s applied in these two positions.

**Figure 6:**
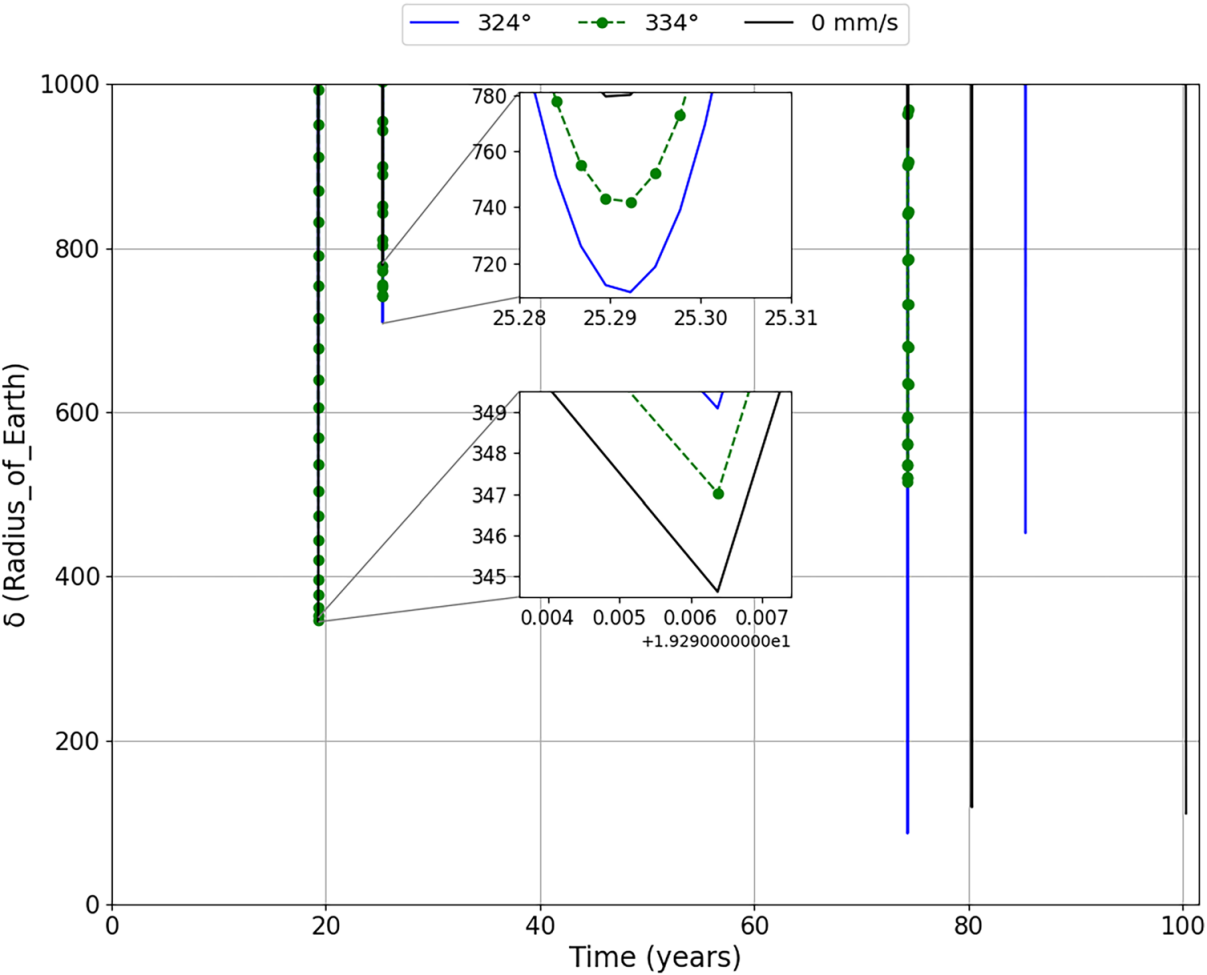
The relative distances between the asteroid and the Earth ($\delta$) when the impulse of −20 mm/s is applied at the mean anomalies of 324° (blue line) and 334° (green dashed line), in June 17, 1980 and June 5, 1980, respectively. We zoomed in on the first two close encounters to better visualize the variations in the approach between the asteroid and the Earth.

Figure 6 shows the behavior of the relative distances between the asteroid and the Earth ($\delta$) for a period of approximately 100 years, for an impulse of −20 mm/s applied in the mean anomalies of 324° and 334°. In Fig. 6 we have enlarged the region surrounding the first two close encounters between the asteroid and the Earth, remembering that we are considering the region in the Figure as the first two encounters within the range of 0 to 1000 radius of the Earth relative distance between the bodies. Other encounters are occurring before those presented, but beyond 1000 Earth radii. We see that these close encounters between the asteroid and the Earth do not appear, because they are at distances outside the scale used, but they should not be neglected in relation to the effect they produce, since we realized that the first close encounter that we are expanding already shows a significant variation in the relative distance between asteroid and Earth if we take into account the impulses applied.

We can see, in Fig. 6, from the very first large encounter, that the asteroid is offset by approximately 5 Earth radii and 2 Earth radii for the impulse applied at the mean anomalies of 324° and 334°, respectively. At the next large encounter, we find that the asteroid is brought closer to Earth at approximately 55 Earth radii for an impulse applied in 324°, and approximately 40 Earth radii for an impulse applied in 334°. After analyzing these regions, we can now look at the Figure as a whole. We see that when the impulse is applied on the mean anomaly of 324°, it brings the asteroid even closer to the Earth, between 70 and 80 years after its application. On December 16, 2054, it is at a distance of approximately 87 Earth radii, which is about 1.44 times the mean Earth-Moon distance.

Our objective here is to study the methods to move the asteroid away from Earth. However, analyzing this closer approach of the asteroid to the Earth in this period, it is clear that the approach did not generate an impact with the Earth, but the asteroid was moved away from the Earth at a considerably safe distance, that is, even with the close approach in 2054 becoming the shortest relative distance between the asteroid and Earth after the impulse in the period studied here. The results show that this close approach can contribute to a largest deflection afterwards. Therefore, we can not rule out the idea that, for a long-term situation, we could bring the asteroid even closer, but in a safe region, and then, through gravitational perturbation, move it away completely in the dangerous period. As for the application of the impulse in the mean anomaly of 334°, we realized that the closest approach of the asteroid to the Earth becomes the first close encounter with the Earth in approximately 19 years, on December 3, 1999, more precisely, at a distance of approximately 347 Earth radii.

**Figure 7:**
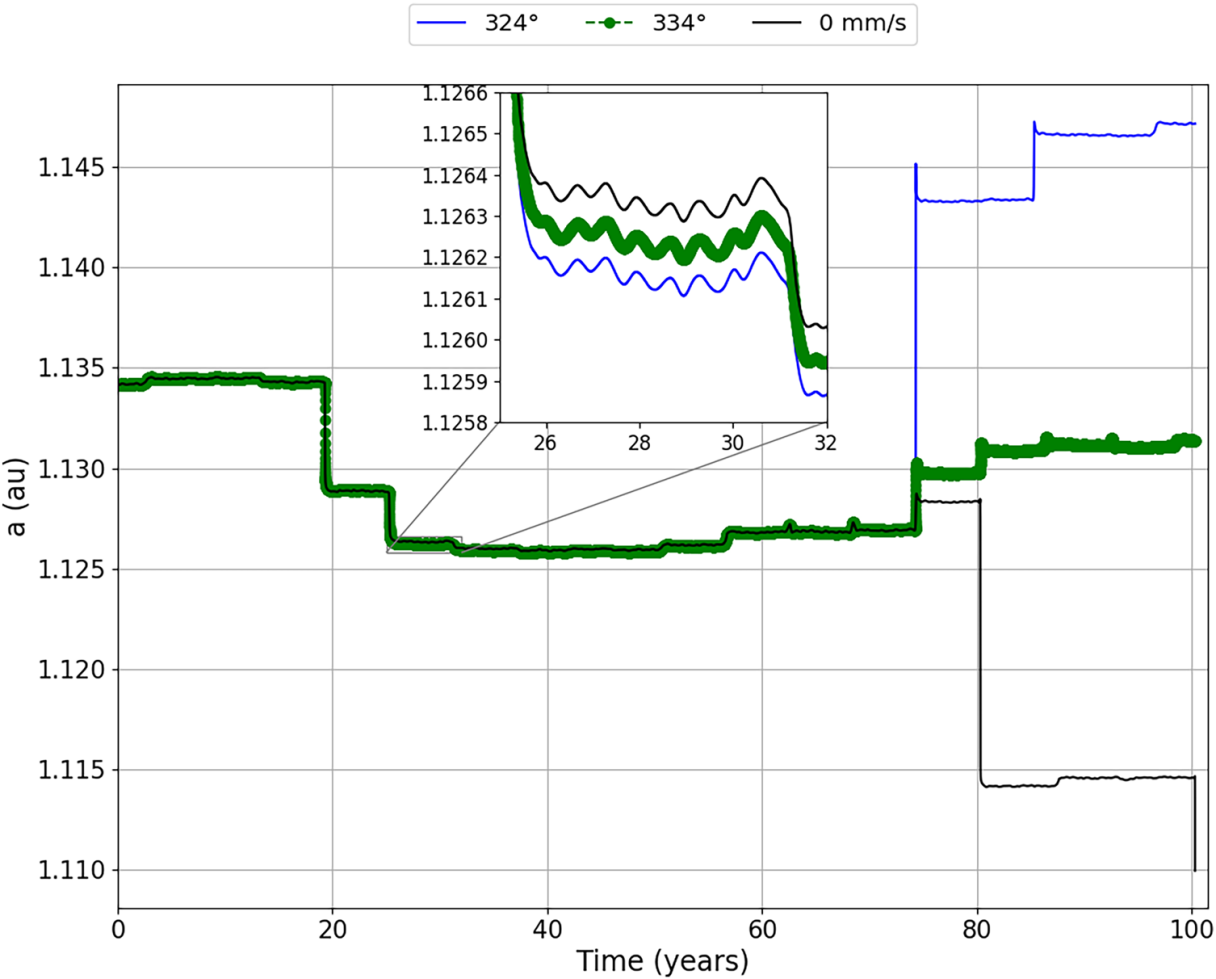
Evolution of the semi-major axis with respect to time for the impulse of −20 mm/s applied at the mean anomalies of 324° (blue line) and 334° (green line). We zoomed in on the region corresponding to the second largest close encounter to better visualize the semi-major axis variations of the asteroid since, at this time, the asteroid has significant variations in its relative distance from the Earth.

**Figure 8:**
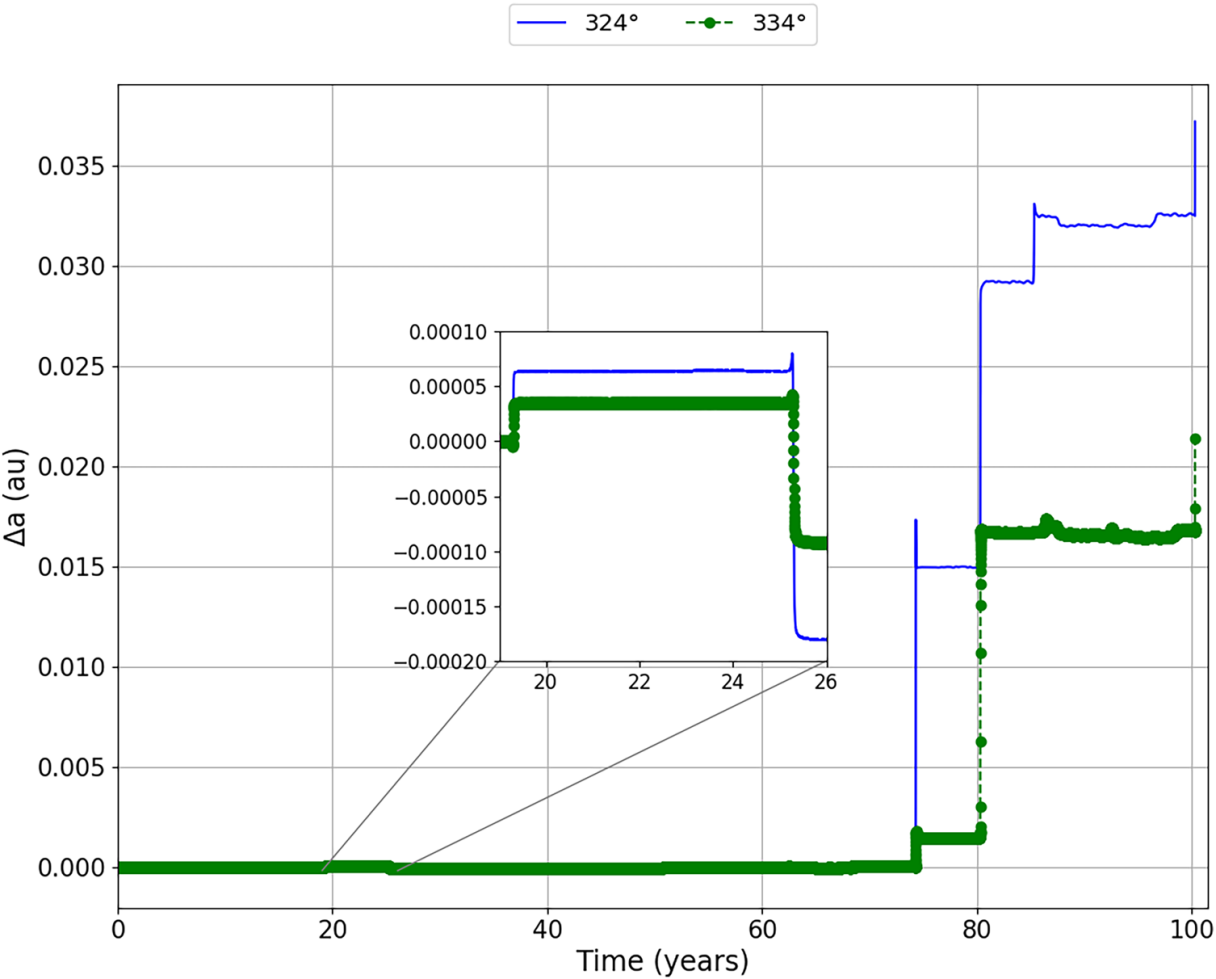
Difference of the semi major axis with respect to time ($\Delta$a) for the impulse applied at the mean anomalies of 324° (blue line) and 334° (green line). We zoomed in on the region corresponding to the second largest close encounter to better visualize the variations of the semi-major axis difference of the asteroid.

Figure 7 shows the evolution of the semi-major axis of the asteroid as a function of time. We found that, when going through the first close encounter, the asteroid naturally undergoes a first gravitational perturbation. This perturbation decreases the semi-major axis of the asteroid until it enters an ‘almost neutral’ region, where we can see that the asteroid remains for a safe period. At this moment the asteroid is making its move away from the closest approach with the Earth. In the second close encounter, where we found a larger variation in the relative distance between the asteroid and the Earth, it is observed that the semi-major axis decreases and then decreases again, but by a smaller magnitude. We enlarged this region to better study the phenomenon. As we can see that, the impulses decrease the semi-major axis with more intensity than the natural phenomenon of close encounters without the application of the impulse. We can see this variation better in Fig. 8, where we show the difference ($\Delta$a) of the semi-major axis with the impulses in relation to the semi-major axis without the impulse.

We can associate the Energy of the asteroid with its semi-major axis. We will notice that, by decreasing the semi-major axis even more, the asteroid loses energy, as it is becoming more and more negative. This variation in energy will make the asteroid to change its position relative to the Earth. Looking at the close approach of the asteroid to the Earth in 2054, provided by the impulse applied at 324°, we will see that large semi-major axis differences occur, where the semi-major axis is increasing considerably, making the asteroid to gain energy. For the impulse applied at 334°, we also found the effect of the energy variation due to the approach of the asteroid to Earth. However, we see that the semi-major axis increase occurs after 80 years after the impulse, making the semi-major axis to remain close to the one just after the previous passage. In other words, the semi-major axis does not decrease drastically, as it naturally does without impulse, nor does it increase as much as in the situation where the impulse is applied at 324°.

An interesting behavior shown in Fig. 7 is be a peak in 2054, with the first major variation that occurs between 70 and 80 years. It can be found both for the impulse applied at 324°, more visible, and for the impulse applied at 334°. Following the evolution of the semi-major axis for the impulse applied at 324°, we can see that the semi-major axis increases with the larger encounter with the Earth, has its highest value made using and then decreases a little until it continues evolving with time. An explanation for the occurrence of this peak can be the two-body problem dynamics. We have that the semi-major axis is calculated through the energy of two bodies, in this case, the energy of the asteroid in relation to the Sun. It depends on the position and velocity of the asteroid relative to the Sun. When the asteroid approaches the Earth, it is accelerated and, when it reaches the perigee, its velocity relative to the Earth is maximum and we have that its velocity relative to the Sun is also maximum, since we have only a sum of vectors. This fact increases the “two-body” energy Sun-asteroid for a short time, so also increasing the semi-major axis of the orbit of the asteroid around the Sun. When the asteroid moves away from Earth again it is decelerated and then it has the general change that we see in Fig. 7, where the semi-major axis is ‘stabilised’ for a while until it has a new encounter with Earth.

**Figure 9:**
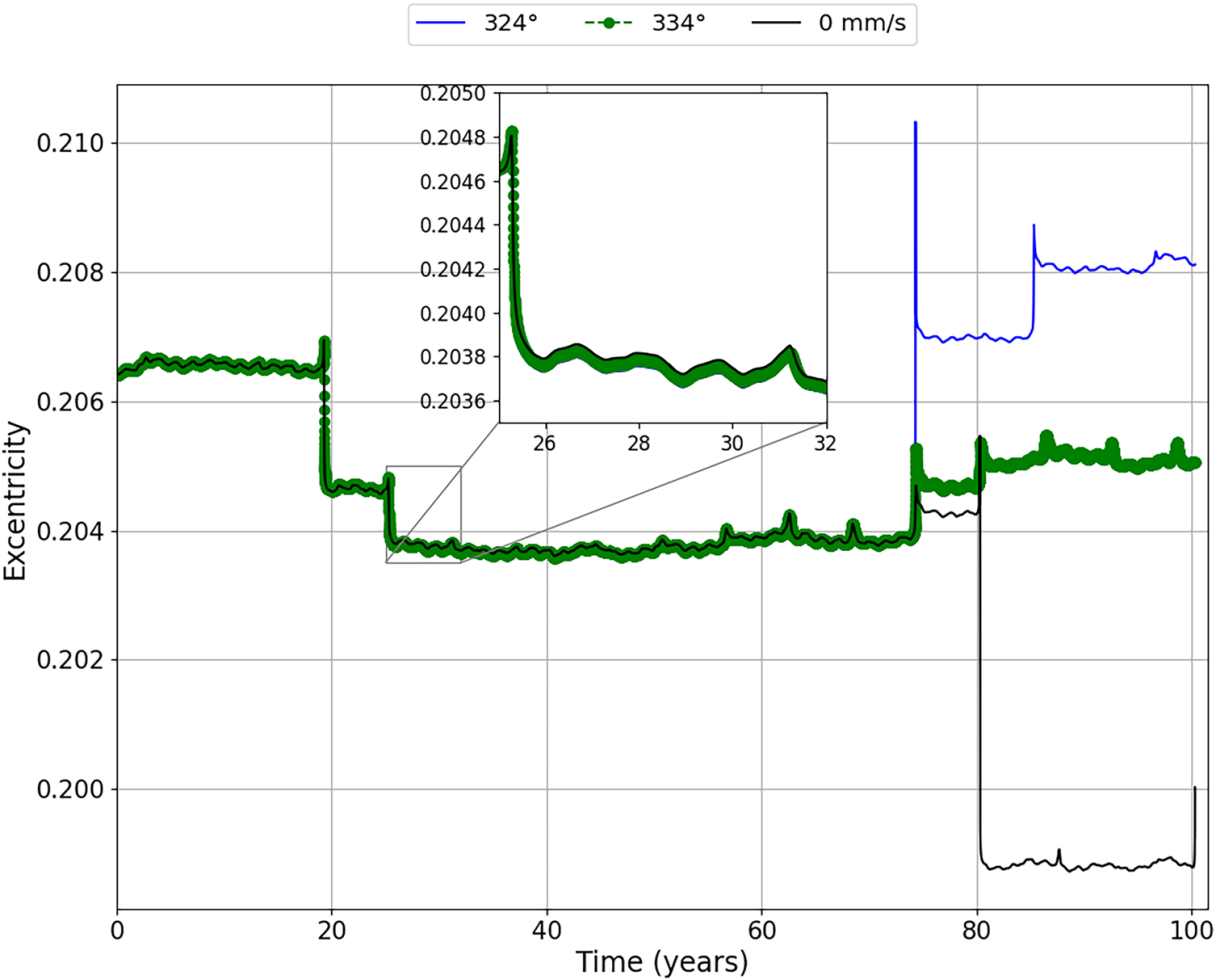
Evolution of eccentricity of the asteroid (e) over time for the impulse of −20 mm/s applied at the mean anomalies of 324° (blue line) and 334° (green line). We zoomed in on the region corresponding to the second largest close encounter to better visualize the variations in the eccentricity of the asteroid, since at this time the asteroid has significant variations in its relative distance from the Earth.

**Figure 10:**
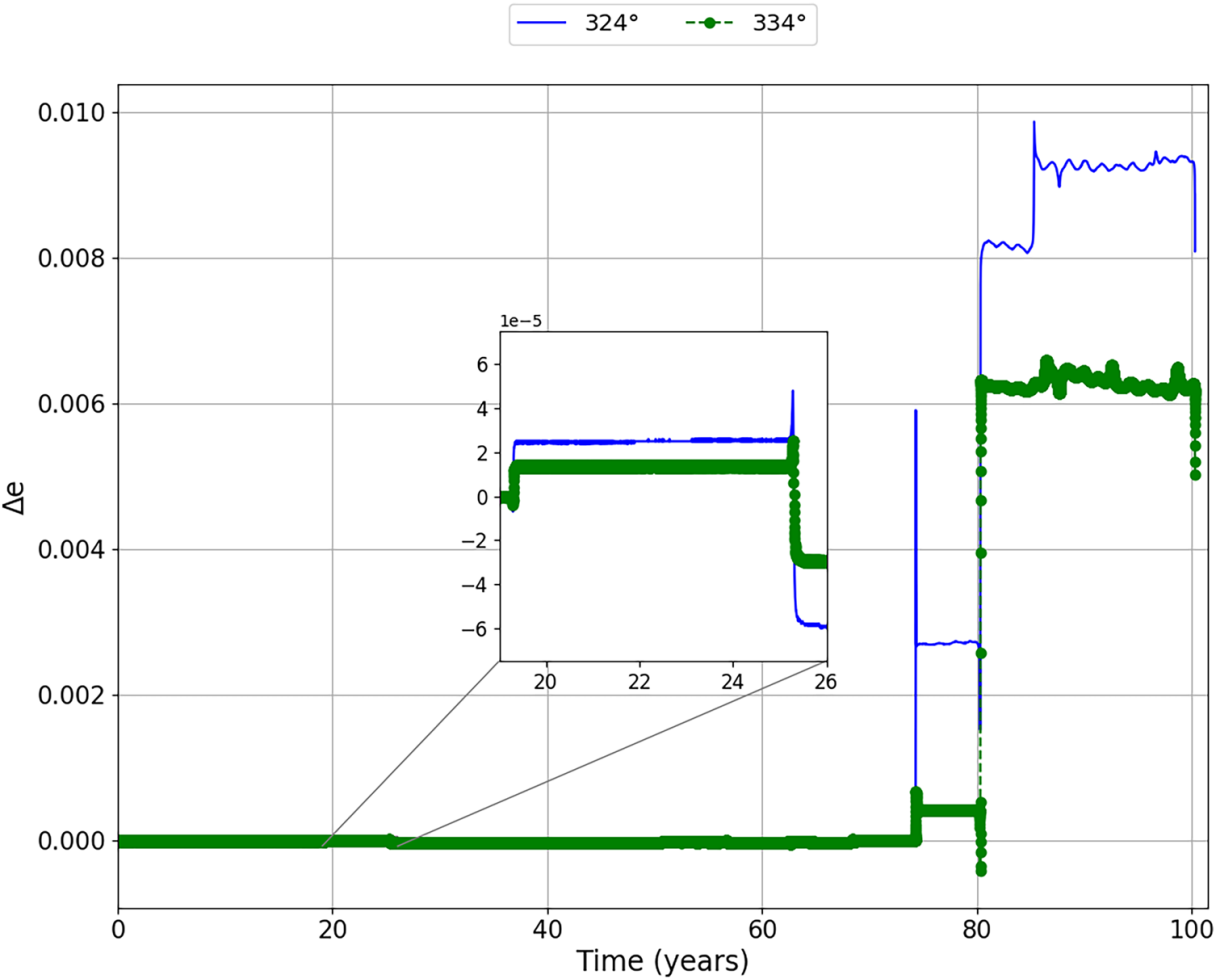
Difference between eccentricity with and without the impulse over time ($\Delta$e) when the impulse is applied at the mean anomalies of 324° (blue line) and 334° (green line). We zoomed in on the region corresponding to the second largest close encounter to better visualize the variations in the eccentricity difference of the asteroid.

Figures 9 and 10 show the evolution of the eccentricity and the eccentricity differences when the impulses are applied. We found variations for the eccentricity in the same moments of the variations of the semi-major axis. We noticed that, in the second close encounter of the asteroid with the Earth, the eccentricity decreases, which makes the velocity differences decrease due to the change in the distance between perihelion and aphelion. However, these variations are very small, in the order of $10^{-5}$. A very different scenario can be found in 2054 where, for the impulse applied at the mean anomaly of 324°, there is a large increase in the eccentricity of the asteroid, which leads to an increase in the velocity difference, since we will have a closer perihelion and the aphelion will be further away. At the next close encounter, we found yet another big increase in eccentricity, but for the two positions of the impulse applied. Furthermore, it seems that the eccentricity related to the impulse applied at 334°, the system enters a cycle of encounters similar to a resonance, which can be stable for longer times. Remember that this cycle presents itself for the observed period, with close encounters approximately every 5 years. However, further in-depth studies are needed.

**Figure 11:**
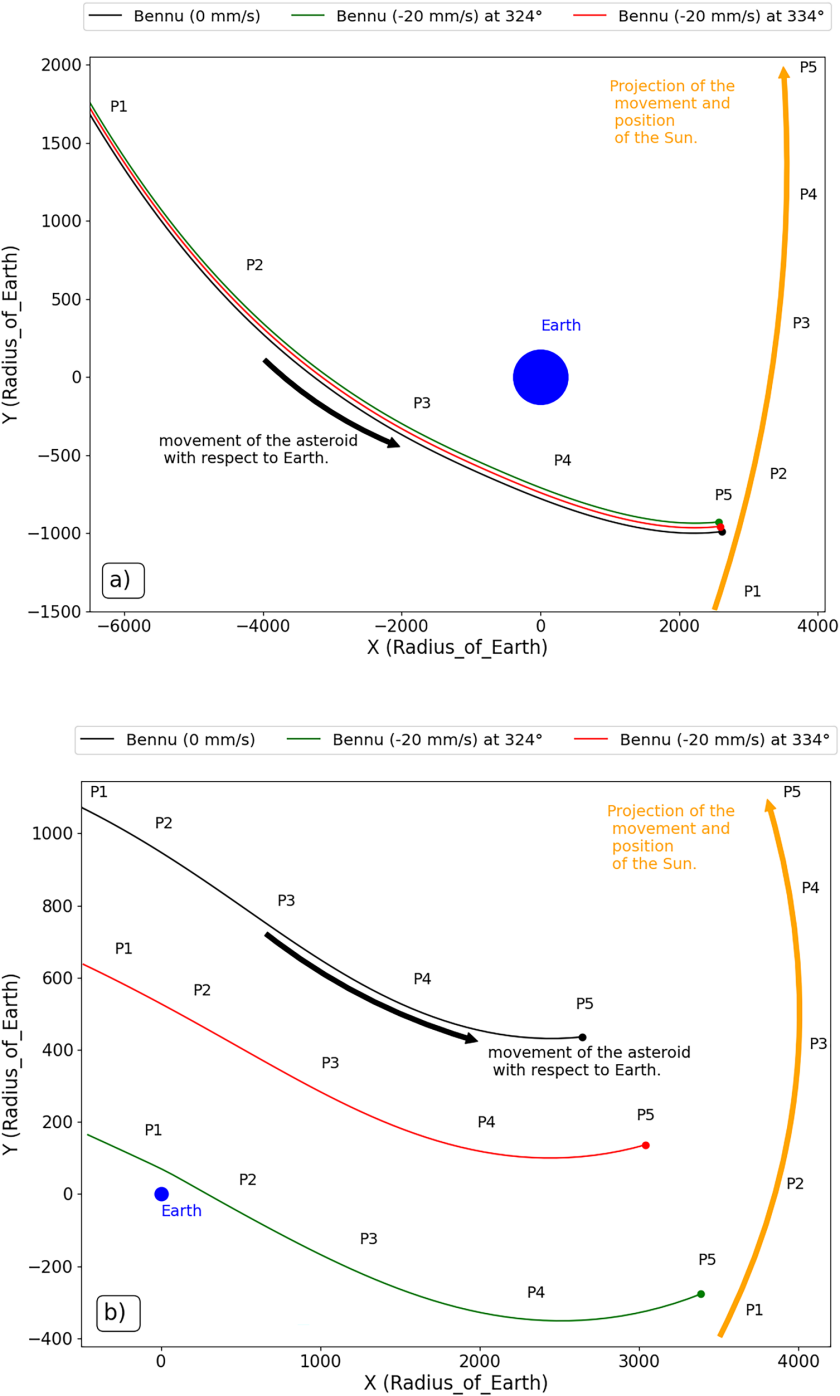
The position of the asteroid relative to the Earth without impulse and with an impulse of −20 mm/s applied at the mean anomalies 324° (green line) and 334° (red line), for the second largest encounter with Earth in 2005, approximately 25 years after the impulse (**a**) and for the largest close encounter between the asteroid and the Earth in 2054, between 70 and 80 years after the impulse (**b**). We call P1, P2, P3, P4 and P5 the relative positions between the asteroid and the Sun, which is shown with respect to the Earth (blue).

These variations in asteroid velocity differences are crucial to understand our results, since, by changing the velocity of the asteroid, we were able to increase its velocity to the point where the asteroid was moved forward when passing by the position that would be the close encounter with the Earth naturally. It approaches Earth, if the Earth is naturally passing by the rendezvous region before the asteroid, or delay the asteroid to the point where it misses the close rendezvous with the Earth, so the Earth passes through the rendezvous point earlier than the asteroid. If the asteroid naturally passes the nearby rendezvous point earlier than the Earth, it will pass closer to the Earth due to this delay. We can see this scenario better in Fig. 11.

In Fig. 11a, we can see the positions of the asteroid in relation to the Earth for a period of 100 days, considering in this time interval the positions before and after the second close encounter that occur in 2005. We also indicate the movement of the Sun in relation to the Earth to better visualize the movement that the asteroid performs due to the gravitational attraction of the Sun. It is already visible in this figure the variations in the approaches that occur between the asteroid and the Earth after the impulses are applied, where we see that the impulse applied at the mean anomaly of 324° brings the asteroid a little closer to the planet. It is also natural to think of this close encounter between the bodies as a ‘Swing By’ between the asteroid and the Earth, where it will vary its energy. At this moment we also notice that the asteroid is a little behind in relation to its natural movement. We can detect this fact by the last position of the asteroid in both parts of Fig. 11 (P5), which was explained by the analysis of the semi-major axis and eccentricity evolution.

Analyzing now Fig. 11b, we found even more visible the gravitational maneuver occurring due to the smaller distance between the bodies. It is easy at this point to see that the asteroid is far ahead of its natural motion at this position, even at the start of the position of the asteroid when the impulse is applied at the mean anomaly of 324°. We noticed that the asteroid already started its position in the time interval (P1) closer to the Earth when compared to its motion without impulse. When the impulse is applied at 324°, the point P1 is very near P2 of the motion without impulse, which we see on a smaller scale for the trajectory corresponding to an impulse applied at 334°. This is also very clear in Fig. 11b. This is even more evident in the last position of the asteroid (P5), when we see that the asteroid is much further ahead than its natural position.

This figure proves our whole idea that, over time, the asteroid makes several ‘Swing By’ with the Earth, changing its energy. These variations change the velocity of the asteroid enough to delay its passage by the closer encounter to the Earth, causing it to either move away or even closer to the Earth. The same condition applies to the case where the asteroid advances its passage by the close rendezvous point with the Earth due to its velocity increase. This is an idea that was already addressed by Carusi et al.^5^, Park and Ross^21^, Ledkov et al.^10^, among others. We are now applying this idea for longer periods and mapping the best positions to apply the impulses by kinetic impact through the M.I.O.A., Figs. 2, 3 and 4, in order to explain the ‘Swing by’ phenomenon within the asteroid diversion scenario.

## Discussion

The results presented here show that the Swing By becomes important in the asteroid deflection technique over a relatively long period, where the position of application of the impulse must be considered. Knowing that, for larger impulses we also need a larger-mass impactor and, for the feasibility of using this deflection method, our results show that, due to the several close encounters of the asteroid with the Earth, we do not really need a large impulse to move the asteroid away, but we need to choose the best positions to apply the impulses. This result has already been mentioned in other works, such as Carusi^5,7^.

We observed that the impulses applied against the movement of the asteroid, in the perihelion of its orbit, have the ability to move the asteroid away from the Earth. The impulse of 10 mm/s showed to be the one with the most effective results when applying impulses in the perihelion of the orbit for the case studied here. However, some applications of impulse in the aphelion of the orbit of the asteroid also showed good results, such as impulses of −10 mm/s and −20 mm/s, which are in the sense of using smaller impactors. We also show, using the M.I.O.A., that we have some other good possibilities along the orbit of the asteroid to make the impulse. For example, if we apply negative impulses to the velocity of the asteroid, for a scenario where we want to move it away by large magnitude, we could apply the smallest impulse studied here among the regions between aphelion of the orbit of the asteroid and the 36° before the perihelion of the orbit of the asteroid. As for positive impulses, we have that this region starts a little before the perihelion and goes up to 55° before aphelion, at the mean anomaly of 135°. We take this result as very encouraging, since they present results that can be used according to the best strategies regarding the planning of possible missions, since we will be able to identify the best position to apply the impulse and also analyze the best scenario, taking into account economics factors. For example, looking for a scenario where the asteroid is not too far from the Earth to apply the impulse, generating economy in the mission. We will not go into detail here, but it is something relevant.

The main point of our work remains to show that using several close encounters between the asteroid and the Earth, it performs a natural Swing By with the Earth, and when we apply impulses in its orbit, the asteroid suffers variations in its relative distance with the Earth that generate variations in its energy, so the asteroid changes its velocity which can both delay or advance a passage that would occur with the Earth in a period of approximately 100 years. This factor is responsible for bringing it closer or away from the Earth. As discussed in section 0.3, we can also bring the asteroid a little closer to Earth, at a safe distance, to deflect it later from an impact risk situation, thus utilizing the larger gravitational perturbation generated by the closest approach to the planet.

## Data Availability

All data generated or analyzed during this study are included in this published article in form of figures.For more information, contact Bruno Chagas Santos (bruno.ba.987@gmail.com or bruno.chagas@unesp.br).
